# Editorial for inaugural issue of *Journal of Brachial Plexus and Peripheral Nerve Injury*

**DOI:** 10.1186/1749-7221-1-1

**Published:** 2006-09-29

**Authors:** Rahul K Nath

**Affiliations:** 1 Texas Nerve & Paralysis Institute, USA

## 

Today marks the publication date of the inaugural issue of the *Journal of Brachial Plexus and Peripheral Nerve Injury *[1]. My immediate thanks goes out to my co-editor in chief, Professor Rolfe Birch, Head of the Royal National Orthopedic Hospital Peripheral Nerve Unit, in London, U.K. Professor Birch is a living encyclopedia of the history and development of peripheral nerve injury management and has been an invaluable personal and scientific resource for guiding the journal from initial planning to the first steps it is taking now.

Our aim is that the journal will be the pre-eminent repository of knowledge for brachial plexus and peripheral nerve injury science. Injury in this context does not imply trauma alone, but any derangement of the anatomy, physiology or biochemistry that results in an altered state of functioning, truly an unlimited scope for a fascinating and important field.

The journal is in open access format, in my view the only relevant such model in these times where information must be freely available to all. The traditional subscription-based model inherently denies free access to knowledge and seems contrary to the spirit of scientific thought. In support of this concept, we have arranged with BioMed Central to subsidize the costs of publication for the first two years of publication. Therefore, there are no barriers to publication of high quality science or to access to this knowledge.

It is very important to note that all submissions, upon approval by our peer review process, are immediately indexed in PubMed and archived in PubMed Central, the full-text repository of the United States National Library of Science. Many other growing indexers such as Google Scholar will also display our articles, making them more widely read and accessible than any other system. Our goal is to have a rapid peer review process, and we will make every effort to make an initial decision within 6 weeks of submission.

The peer review process will match knowledgeable reviewers with submitted manuscripts to produce high quality articles of interest and scientific merit. The process is confidential so that criticisms and revisions are made in the fairest manner possible. The final decision on publication will be made by the editors in chief.

We will look for submissions of interesting and important scientific information that hopefully will have clinical application. This focus does not deny the relevance of basic research, it embraces it: the future of nerve injury management lies in manipulation of molecular mechanisms, less so in classic surgical and medicinal treatments. Whether there are obvious clinical implications or not, high quality research of all types will be nurtured and presented with pride in the journal.

One of the main goals of our endeavor is to cultivate cross disciplinary dialogues to enhance our understanding of mechanisms and to develop strategies for treatment of various disorders. As an example, a neurologist may be interested to know the potential application of surgical techniques such as nerve transfer to upper motor neuron lesions like stroke that are not fully treatable with medicines; conversely, surgeons should be aware of potential sensory and motor imbalances typically see by neurologists which may be candidates for surgical treatment. And both groups will enhance their management of patients by knowledge of breakthroughs in underlying molecular mechanisms.

Prior to today's inauguration *Journal of Brachial Plexus and Peripheral Nerve Injury*, there has been no single, dedicated place to discuss these critical issues. We have the opportunity to improve the quality of life of so many people in developing countries and otherwise disadvantaged situations. The talent and determination of our readers is a resource with unlimited potential and we have this as a motive to produce and foster new scientific thought for the benefit of the community affected by brachial plexus and nerve injury. This is the most exciting concept of all.

I look forward to a most interesting future for *Journal of Brachial Plexus and Peripheral Nerve Injury *and for the International Society for Brachial Plexus and Peripheral Nerve Injury [2]. Our editorial board includes world leaders in all areas of our field and a comprehensive international distribution. There is every reason to expect that we will set new standards for the study of peripheral nerve injury and in the process improve public health significantly. I thank our editorial board and our contributors and readers for their interest and efforts in advancing this worthy cause.
